# Theories of Sex

**Published:** 1910-07-23

**Authors:** 


### THEORIES OF SEX.

A recent experiment, in which frogs' eggs were made to undergo segmentation and develop by the stimulus of a needle-prick, may possibly cause a revision of our views on the origin of sex. Many years ago Professor Balfour is reputed to have said that there seemed to be no adequate reason why the ovum should not develop without the assistance of the spermatozoon. Some authorities have declared that they believe in polyspermy, and so far as concerns the impregnation of the human ovum there is no direct observation to disprove this theory, though on comparative analogy it is held to be erroneous. There is again another body of experimenters who have been for some time endeavouring to apply quasi-Mendelian theories to sex, and have met with a certain measure of success. The disadvantage of this method is that it deals with almost ultra-microscopic entities, and although Professor Wilson published an interesting *résumé* of the current theories, the exceptionally delicate nature of an investigation into the number and peculiar constitution of chromosomes renders the work somewhat hazardous in result. Moreover, in all these cases of so-called simple Mendelian inheritance the pyschological side of the question is generally overlooked, and although it may be possible to predict the colour of the " get " of a certain bull when mated with certain cows, his qualities of character may be inherited and lost according to totally different propositions.

There is in chemical substances which contain a carbon atom combined with four different groups a peculiarity which bears a curious resemblance to the facts noticeable in the case of sex. This combination of one carbon atom with $R_1$ $R_2$ $R_3$ $R_4$ can exist in two different modifications, called dextro and laevo, which are optically active. But if the two are produced together in equal quantities the result is a " racemic " compound $d+l$, which is optically inactive. It happens that whenever chemists artificially synthesise a compound containing asymmetric carbon atoms the result is always a racemic compound, whereas the naturally occurring compound is in most cases either "d" or " l." It seems, therefore, that the probabilities in nature for " d " and " l " are about equal. Certain enzymes have the power to attack one and leave the other, so that a synthesised racemic compound can generally be made to yield an optically active compound by treatment with an appropriate enzyme. This is, of course, a brief statement of the discovery made by Pasteur about the year 1860, but it suggests new possibilities with regard to the solution of the sex problem. The human ovum, or for that matter any ovum, possesses a certain chemical composition. The spermatozoon also can be considered chemically. Then it may be suggested either that the ovum contains, so to speak, both " d " and " l " ingredients, and that the action of the spermatozoon is really that of an enzyme, or that among animals the male represents " d " and the female " l," or *vice versa*, and that the union of the two produces a chemically active body, which proceeds to develop on certain lines. The idea that the spermatozoon is enzymic in nature seems to fit the known facts about the nature of the ovum more satisfactorily. The two sexes then correspond to the " d " and " l " of the natural carbon compound, and the probabilities should be equal. This, however, seems to be disproved by the fact that males seem to be born in a number which preponderates slightly over the female. Theories which concern the size of the ovaries or the periods of menstruation seem doomed to failure from the outset, since they do not go back far enough. The idea that the sex of the egg is predetermined need not necessarily be correct: the act of fertilisation may be the only determining cause. In view of the tadpole experiments it may be suggested that the prick of the needle produces a sufficient rearrangement of atoms to ensure activity, for even with so small a lesion something must inevitably be lost.

